# Phenotypic and genotypic characterization of acaricide resistance in *Rhipicephalus microplus* field isolates from South Africa and Brazil

**DOI:** 10.1016/j.ijpddr.2023.100519

**Published:** 2023-12-27

**Authors:** Dieter J.A. Heylen, Michel Labuschagne, Christina Meiring, Luther van der Mescht, Guilherme Klafke, Livio Martins Costa Junior, Tom Strydom, Jeanette Wentzel, Caryn Shacklock, Lénaig Halos, Francois Maree, Josephus Fourie, Maxime Madder, Alec Evans

**Affiliations:** a Evolutionary Ecology Group, Department of Biology, University of Antwerp, Wilrijk, Belgium; b Interuniversity Institute for Biostatistics and statistical Bioinformatics, Hasselt University, Diepenbeek, Belgium; c Clinomics, P.O. Box 11186, Universitas, Bloemfontein, 9321, Uitzich Road, Bainsvlei, Bloemfontein, 9338, South Africa; d Clinglobal, B03/04, The Tamarin Commercial Hub, Jacaranda Avenue, Tamarin, 90903, Mauritius; e Clinvet International Pty (Ltd), Uitzich Road, Bainsvlei, Bloemfontein, 9338, South Africa; f Department of Zoology and Entomology, University of the Free State, Bloemfontein, South Africa; g Instituto de Pesquisas Veterinárias Desidério Finamor, Centro de Pesquisa em Saúde Animal, Estrada do Conde 6000, Eldorado do Sul, RS, 92990-000, Brazil; h Laboratory of Parasite Control, Federal University of Maranhão, Sao Luís, MA, Brazil; i MSD ANIMAL HEALTH, Private Bag X2026, Isando, 1600, South Africa; j Hans Hoheisen Wildlife Research Station, Department of Veterinary Tropical Diseases, University of Pretoria, South Africa; k Afrivet, Hazeldean, South Africa; l Bill & Melinda Gates Foundation, Seattle, WA, USA; m Clinvet International Pty (Ltd), 1479 Talmadge Hill South, Waverly, NY, 14892, USA

**Keywords:** Acaricide, Resistance, *Rhipicephalus microplus*, Africa, Brazil

## Abstract

*Rhipicephalus* (*Boophilus*) *microplus* is one of the most successful ticks infesting cattle around the world. This highly-invasive species transmits cattle parasites that cause cattle fever leading to a high socio-economic burden. Tick eradication programs have often failed, due to the development of acaricide resistance. Here we characterize acaricide resistance in a large number of tick isolates from regions in South Africa (KwaZulu Natal, Mpumalanga, Western & Eastern Cape provinces) and two Brazilian regions.

By means of Larval Packet Tests (LPT's) acaricide resistance was evaluated against five commonly used acaricides (chlorfenvinphos, fipronil, deltamethrin, amitraz, and ivermectin). Furthermore, the coding region containing the knock down resistance (*kdr)* mutation, known to result in pyrethroid resistance, was sequenced.

Resistance to at least one acaricide class was reported in each of the five regions, and a high proportion of tick isolates exhibited multi-resistance to at least two acaricide classes (range: 22.2–80.0%). Furthermore, resistance ratios (RR) showed high spatial variation (intercontinental, as well as regional) but low regional spatial autocorrelation. Previous and current acaricide use correlated with current RR, and several combinations of acaricide RR were positively correlated. Moreover, fipronil resistance tended to be higher in farms with more intense acaricide use. The *kdr*-mutations provided the ticks a fitness advantage under the selection pressure of synthetic pyrethroids based on population (*kdr*-allele frequency) and individual level data (genotypes).

The data show the threat of acaricide (multi-)resistance is high in Brazil and South Africa, but acaricide specific levels need to be assessed locally. For this purpose, gathering complementary molecular information on mutations that underlie resistance can reduce costs and expedite necessary actions. In an era of human-caused habitat alterations, implementing molecular data-driven programs becomes essential in overcoming tick-induced socio-economic losses.

## Highlights

• The invasive *Rhipicephalus microplus* transmits virulent parasites in cattle.
• Development of acaricide resistance becomes a global problem.
• Bio-assays reveal highly prevalent multi-resistance in South Africa and Brazil.
• Genetic mutations result in acaricide resistance, like *kdr*-mutations.
• Low spatial auto-correlations ask for local resistance assessments.

## Graphical abstract

**Figure:**
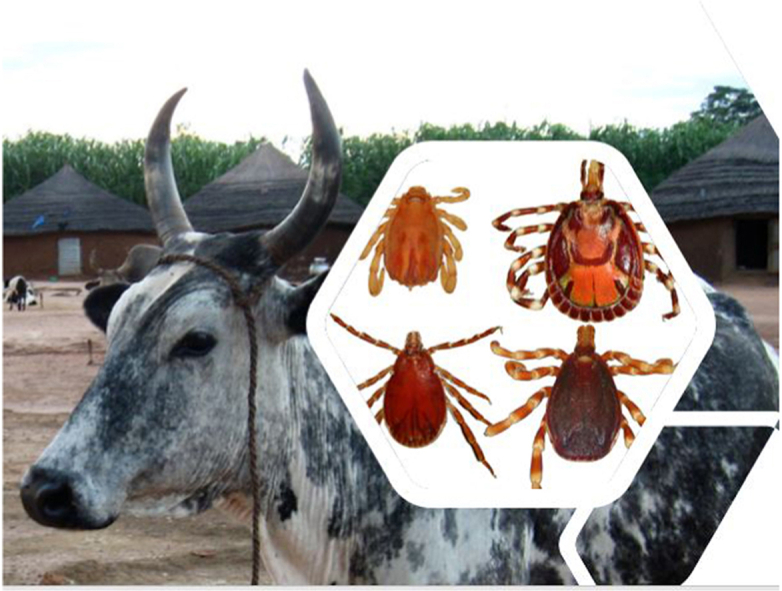

## 1 Introduction

The majority of the human African population lives in rural areas where they heavily rely on agriculture, including livestock production (Jahnke, 1982). Many Sub-Saharan farmers belong to resource-constrained farming communities and struggle to maintain minimal life standards, often due to the harm caused by ecto- and endoparasites, including (invasive) vector-borne infectious diseases (Young et al., 1988; Jongejan and Uilenberg, 2004). Among those parasites affecting livestock, ticks have the most significant impact through both direct effects as well as those resulting from the diseases they transmit. Direct constraints include, but are not limited to, reduced weight gains, lower growth rate, reduced nutrient utilization, lower meat and milk yield, reduced value of hides, tick paralysis and the relieve of individual animals suffering by culling. Indirect constraints include transmission of some of the most important livestock diseases: anaplasmosis (gallsickness), babesiosis (red-water), theileriosis (East Coast fever) and heartwater (Jahnke, 1982; Coetzer and Tustin, 2005; Heylen et al., 2023). The effective control of ticks and tick-borne pathogens is mainly done using acaricides. However, with the widespread development of resistance to different classes of acaricides, there is a need for novel acaricides and innovative livestock tick control programs and methods that can integrate with existing methodologies (Jongejan and Uilenberg, 2004; Abbas et al., 2014; Rodriguez-Vivas et al., 2018).

Similar needs occur in regions of higher welfare, where a substantial part of the economy relies on the success of the cattle industry. Brazil has the world's second-largest national cattle herd, and is the world's largest exporter of beef. The United States Department of Agriculture projects that Brazil will continue its export growth trajectory for the next decade, reaching 2.9 million metric tons, or 23 percent of the world's total beef exports, by 2028 (Zia et al., 2019). Heritable resistance against multiple acaricides has been detected in *Rhipicephalus* (*Boophilus*) *microplus* ticks infesting Brazilian cattle, which is a dramatic outcome given the limited number of compounds available (Jongejan and Uilenberg, 2004; Klafke et al., 2017).

Detection methods of acaricide resistance in an early stage are essential, allowing for the evaluation of the use of an effective chemical before applying it in control strategies, and hence avoid further selection for and spread of resistant phenotypes. Hereto, Food and Agriculture Organisation (FAO) has recommended bio-assays that can be done at low costs: adult immersion test, and Larval Packet Test (LPT) evaluating tick survival after exposure to an acaracide (FAO, 2004). These assays are very labour-intensive, require live ticks of a specific age including a susceptible reference strain, are more difficult to execute in remote areas, and cannot generate the required data within a short time frame.

Acaricide resistance is widely acknowledged to be inherently linked to the tick's physiology at the molecular level (Feyereisen et al., 2015; De Rouck et al., 2023). A comprehensive understanding of the genetic basis underlying resistance physiology is described by Feyereisen et al. (2015), and for a recent review of the molecular mechanisms of acaricide resistance De Rouck et al. (2023) can be consulted. Non-harmful mutations that partly describe the genotypic resistance profile of a tick (or a population of ticks) ideally should be readily detected as molecular markers. Still, fundamental genomic research has rarely been translated into practical diagnostic assays that support the rapid detection of acaricide resistance enabling decision making at a farm level (Rodriguez-Vivas et al., 2018).

Our study focuses on acaricide resistance in the tick *Rhipicephalus microplus*, one of the most successful cattle ticks in the world. It is a remarkable species because of its invasive behaviour into (sub-)tropical regions. It has been introduced from the bovid- and cervid-inhabited forests of the Indian region to many areas of tropical and subtropical Asia, northeastern Australia, Madagascar, coastal lowlands of southeastern Africa to the equator, and much of South and Central America, Mexico and the Caribbean (Estrada-Pena et al., 2006 and references herein). After its introduction in West Africa in 2004–2007, the tick spread efficiently in the West African region and displaced the local one-host ticks (Madder et al., 2007, 2011, 2012; Adakal et al., 2013). Further spreading was noticed to Nigeria in 2014 (Kamani et al., 2017) and to Cameroon in 2016 (Silatsa et al., 2019). The same time, *R. microplus* expanded its distribution in other parts of Africa, including Angola, Uganda and Kenya, and displaced *R. decoloratus* in several countries like Tanzania (Lynen et al., 2008; Gomes and Neves, 2018; Kanduma et al., 2020; Muhanguzi et al., 2020) and parts of South Africa (Nyangiwe et al., 2017; Van Dalen and Jansen van Rensburg, 2023.

The current distribution of the acaricide resistant phenotypes of this economically important tick is uncertain - largely due to the limitations mentioned above - however efforts to characterize this on a global level have been initiated (Dzemo et al., 2022). Spatial knowledge on the fitness of (resistant) tick isolates in the presence of acaracide use, in addition to emigration and immigration rates, are essential for risk assessments and implementation of effective control measures for ticks and the pathogens they vector (i.e. establishment of proper treatment strategies and prevention). Furthermore, the level of susceptibility of hosts on which ticks potentially feed, as well as tick exposure risks in the community of potential hosts, are basic inputs for (eco-) epidemiological models, but hard to obtain.

The goal of our study was twofold: (1) obtain an up-to-date surveillance of isolates from areas with acaricide resistance in South Africa and Brazil, and link this information to relevant meta-data (current and previous acaricide use), (2) investigate the correlation between the phenotypic resistance and molecular information (knock-down resistance or *kdr*-mutations, see below) and this at individual and population level (cf. Rodriguez-Vivas et al., 2012), For (1), LPT-resistance ratios (RR) were obtained using data on the larval mortality (LPT's) when exposed to one of the five classes of acaricides: organophosphates (chlorfenvinphos), phenylpyrazoles (fipronil), synthetic pyrethroids (deltamethrin), amidines (amitraz), and macrocyclic lactones (doramectin) (Table 1). For (2), we link mortality information to the *kdr*-locus in the voltage gated sodium channel gene (PCR). Resistance against Type II pyrethroids (one of the most widely used pyrethroids in tick treatment) has been described as a knock down resistant phenotype. They prevent the closure of the para-sodium channel blocking synaptic transmission causing flaccid paralysis. Knock down resistant phenotype mutants reduce the affinity for the pyrethroid binding site of the channel reducing its effect (Cossio-Bayugar et al., 2020).

**Table 1:** Acaricide classes and concentrations (mg/mL) for larval packets. LC_50_ (Lethal Concentration, 50%), representing the concentration of the acaricide that is expected to cause the death of 50% of the tick population, are provided for the susceptible strains of South Africa and Brazil.

Class	Amidine	Organophosphates	Synthetic pyrethroids	Macrocyclic lactones	Phenylpyrazoles
Compound	Amitraz	Chlorfenvinphos	Deltamethrin	Doramectin	Fipronil
Dilution 1	0.20	0.20	0.500	4.00	4.00
2	0.10	0.13	0.125	2.60	0.80
3	5.00 × 10^−2^	8.50 × 10^−2^	3.100 × 10^−2^	1.69	0.16
4	2.50 × 10^−2^	5.50 × 10^−2^	7.800 × 10^−3^	1.10	3.20 × 10^−2^
5	1.25 × 10^−2^	3.60 × 10^−2^	2.000 × 10^−3^	0.71	6.40 × 10^−3^
6	6.20 × 10^−3^	2.30 × 10^−2^	4.900 × 10^−4^	0.46	1.28 × 10^−3^
7	3.10 × 10^−3^	1.50 × 10^−2^	1.200 × 10^−4^	0.30	2.56 × 10^−4^
Control	0	0	0	0	0
LC_50_ – South Africa	0.03	0.05	0.04	1.68	0.02
LC_50_ - Brazil	0.02	0.13	0.15	1.23	0.02

## 2 Materials and Methods

### 2.1 Study design and selection of collection sites

To characterize the phenotypic resistance via the standardized LPT protocol (see below) and to evaluate the performance characteristics of ARDA ©, field collections of *R. microplus* isolates were performed. Ticks were collected from farms, dip tanks and pastoralists. At least twenty-five engorged female ticks were collected from multiple animals with each isolate being defined as originating from animals in biogeographically distinct areas. If several herds were co-grazing on community land, the ticks collected from these animals were defined as a single isolate. Engorged adult females were pooled and allowed to oviposit. At approximately two weeks after eclosion, larvae originating from the same pool were either used in the larval packet test or stored in 70% ethanol for subsequent molecular analysis.

**Fig. 1:**
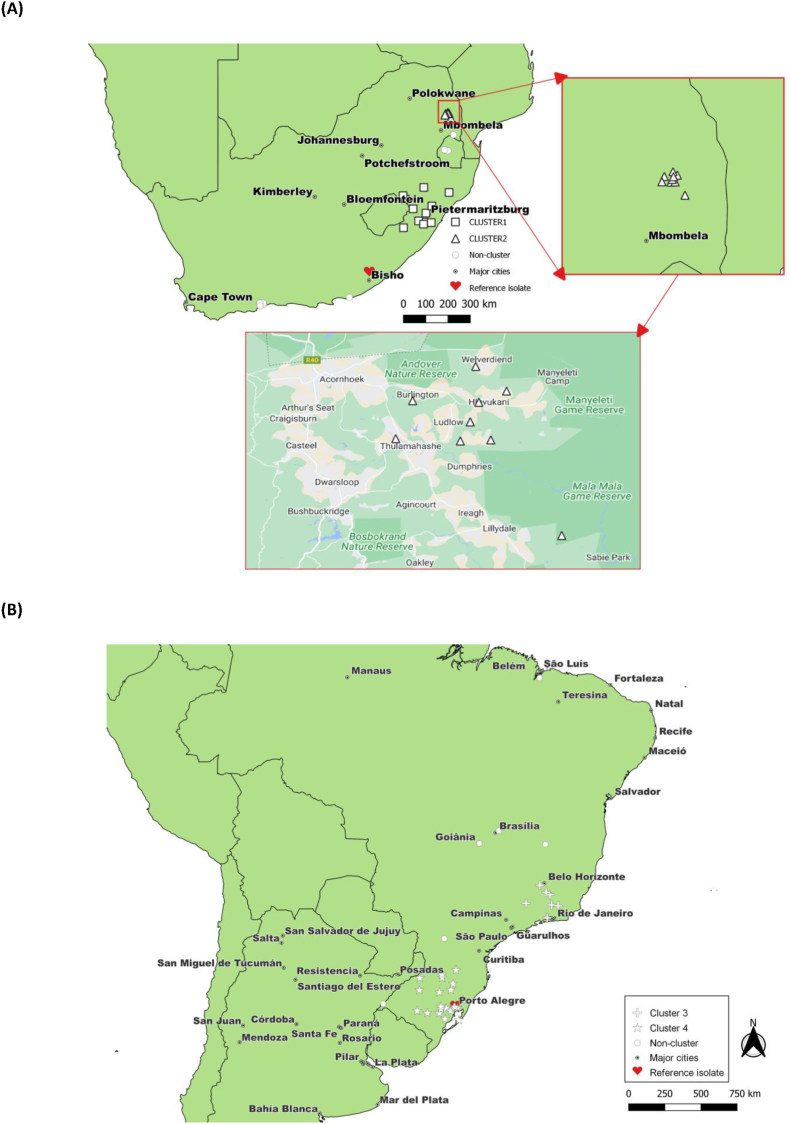
Overview of the sampling locations in (A) South Africa and (B) Brazil.

Five groups partook in the survey (three from South Africa, two from Brazil; see Fig. 1 for sampling sites):

**Lab 1 – South Africa** (KwaZulu Natal, by Afrivet): Collected from rural community dip tanks and commercial farms in KwaZulu Natal. No selection of sites was based on acaricide resistance history, although the team had previously worked with these farms.
**Lab 2 – South Africa** (Mpumalanga, by Hans Hoheisen): All isolates were collected from rural community diptanks of the Mnisi Community program, Bushbuckridge East, Mpumalanga, South Africa. Isolates were collected from all 25 dip tanks within the 5 wards. Environmental officers and the local State veterinarian assisted with the collection of the isolates. The diptanks vary between 400 and 2000 animals per diptank, some were next to protected areas, while others were within the community. The animals were sampled at the state vet diptanks.
**Lab 3 - South Africa** (Western & Eastern Cape provinces, by MSD): Sourced from commercial farms across South Africa (primarily Eastern Cape, Western Cape, but also Free State and Swaziland) as part of a surveillance program. Ticks were collected during routine farm visits by the MSD agents.
**Lab 4 – Brazil** (by IPVDF, Eldorado do Sul, RS - Laboratory of Parasitology): Isolates collected from farms primarily in Southern Brazil: Rio Grande do Sul, but also Paraná, Santa Catarina, Goiás, Federal District Brazil, Minas Gerais and Rio de Janeiro. Isolates were shipped to the facility as part of routine acaricide testing associated with reports of treatment failure in the field.
**Lab 5 – Brazil** (by Federal University of Maranhão - Laboratory of Parasites Control): Isolates collected from farms primarily in Maranhão, Northern Brazil. Isolates were shipped to the facility as part of routine acaricide testing associated with reports of treatment failure in the field.

In order to obtain relevant meta-data on each isolate a questionnaire was completed to obtain the following information: Owner details, GPS coordinates, date and time of collection, history of acaricide use in the herd including products or acaricide classes, frequency of treatment, and the date of the last treatment.

### 2.2 Larval packet test

Dose response assays were carried out according to the modified larval packet test (LPT) as recommended by the FAO (Stone and Haydock, 1962; FAO, 2004). All packets used in the survey were sourced from a single laboratory (TBD International). The packets were prepared with five different acaricide classes and seven concentrations for each class (Table 1). Each tick isolate was ideally tested in triplicate for each concentration, however, due to constraints on the number of larvae available some isolates were only tested in duplicate. Briefly, approximately 100 larvae (taken randomly from the pool) were introduced into packets and incubated after sealing. Larval viability was assessed at 24 h, except for amitraz which was assessed after 48 h. Larvae were considered alive if they were able to walk. Larvae that were able to move their appendages but could not walk were not considered alive.

As per the FAO guidelines, mortality in the control packets was used to adjust mortality in the treated packets. If mortality was <5 %, the direct mortality figures were used to calculate the LD_50_. If mortality was between 5 and 10%, the percentage of mortality in all the groups was corrected using the Abbott's formula: Corrected percentage mortality = 100 × (% test mortality - % control mortality)/(100 - % control mortality). If mortality in the control group was >10%, the test results were considered invalid, and the results were discarded.

Probit analysis was performed to calculate the concentration lethal to 50% (LC_50_) of the larvae.

To calculate the resistance ratios (RR), the LC_50_ values calculated for each of the field isolates were compared to LC_50_ values from characterized susceptible isolates which were determined independently by each of the collaborators. The RR for all South African isolates were calculated based on the same susceptible *R. microplus* isolate originating from South Africa (see Table 1 for LC_50_) whilst all RR for Brazilian isolates were based on a Brazilian susceptible isolate from Porto Alegre (see Table 1 for LC_50_).

Various arbitrary criteria have been proposed to evaluate the resistance level of *R. microplus* to different acaricides, as reviewed by Rodriguez-Vivas and colleagues (Rodriguez-Vivas et al., 2018). Here, a classification was made based on the following RR cut-offs: ‘Susceptible’ (RR < 3.0); ‘Intermediate resistance’ (RR 3.0–26) and ‘High resistance’ (RR > 26).

### 2.3 Quantification allele frequencies at population level

Pools of 25 larvae randomly selected from untreated isolates used in the LPT assays were subjected to homogenization using 2 mm diameter high density ceria stabilized zirconium oxide beads (Beads International, Krugersdorp, South Africa), followed by DNA isolation using the GeneJET Genomic DNA Purification kit (Themo Fisher Scientific, Waltham, MA, USA). Primers KDR_locus-Exon-4F (5′-CTACGTGTGTTCAAGCTAGCCAAATCG-3′) and KDR_locus-Exon-2R (5′-GTTTACTTTCTTCGTAGTTCTTGCC-3′) were barcoded and used to amplify a 172 bp amplicon from DNA isolated from pooled or individual larvae (see below) using Platinum™ SuperFi II PCR 2 x Master Mix according to the manufacturer's recommendations. Thermal cycling entailed initial denaturation at 98 °C for 1 min followed by 30 cycles of 98 °C for 10 s, 60 °C for 10 s and 72 °C for 10 s, with a final elongation step at 72 °C for 5 min. The barcoded PCR products were subjected to library preparation using the LSK109 kit and sequenced using Oxford Nanopore Technologies Minion 9.4 flow cell. Nanopore signals (≥q10) were base called using Guppy 5.0.11 with the super high accuracy (SUP) model to generate sequence data, followed by demultiplexing and binning of the reads using miniBarcoder (Srivathsan et al., 2019). The binned reads were mapped using Minimap2 (Li, 2018) against the reference sequence. Population level *kdr*-percentages were calculated as the percentage of *kdr*-mutation reads per pool analysed. The abovementioned procedure applied to three separate pools of 25 larvae from the South African reference strain, showed that in one case 1% *kdr* was found, and in two cases the pools were found to be >98% wild type.

### 2.4 Genotypes at individual level (KDR/KDR, WILD/WILD, WILD/KDR)

The genotypes of individual larvae from packets treated with deltamethrin were determined by separating surviving and dead larvae at each of the concentrations at the time of assessing viability. These larvae were then subjected to individual sequencing. Barcoded primers KDR_locus-Exon-4F and KDR_locus-Exon-2R were used to amplify DNA from individual tick larva that were homogenized using zirconium oxide beads in a 20 μl reaction volume containing 1 x TE buffer and 0.8 units proteinase K. Homogenized samples were incubated at 56 °C for 5 min and 98 °C for 5 min, resulting in crude extracts to serve as template for PCR amplification (as described above) followed by sequencing. The individual genotypes were generated based on the number of KDR/WILD reads originating from the DNA extracted from each individual larva.

### 2.5 Statistical analysis

**Fig. 2:**
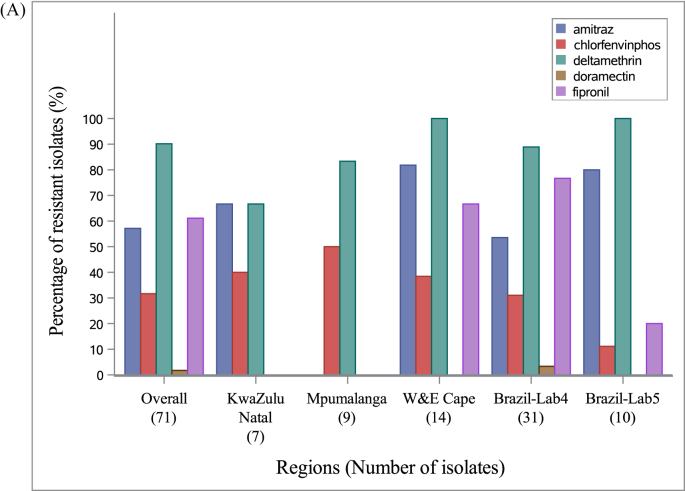

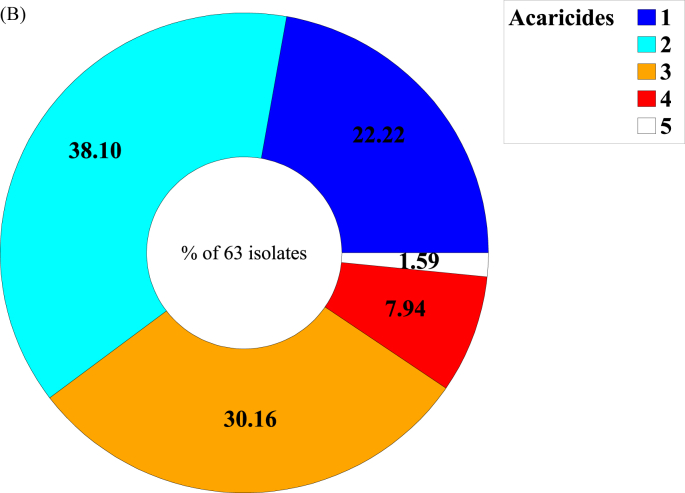
**(A)** Resistant isolates (i.e. showing RR > 3.0 for at least one acaricide) by active ingredient. **(B)** Proportions of single and multi-resistance within the subpopulation of resistant phenotypes in the overall set of isolates considered. As for both KwaZulu Natal (lab 1) and Mpumalanga (lab2) bio-assays for doramectin and fipronil were not executed, multi-resistance is likely underestimated. Details on collection sites (including the Brazilian labs) are provided in the ‘Materials and Methods’ section.

The data possess a hierarchical structure with tick isolate (value RR for each acaricide [0-∞]) nested within a macrogeographic region. Generalized Estimation Equation models were fitted onto the data (see Molenberghs and Verbeke, 2005) taking into account the statistical dependence of observations within region by adding an exchangeable working correlation at this sublevel. The residuals for RR were assumed to follow a normal distribution (identity-link). For the effects of acaricide use on resistance, RR were included as response variables in models with the following explanatory variables: acaricide (used vs. not used), number of acaricide used locally on the farm, time since last acaricide use. When considering the effects of the local use of specific acaricides (current and previous use) on the RR, we evaluated the following combinations, given the shared molecular mechanistic pathways: amidine type acaricides on amitraz resistance; macrocylic acaricides on doramectin resistance; organophosphate acaricides on chlorfenvinphos resistance; phenylpyrazole on fipronil resistance; pyrethroid on deltamethrin resistance. For all analyses, a stepwise backward selection procedure was used to select the best model. At each step we excluded the fixed factor with the highest non-significant P-value (P > 0.05), re-ran the model and examined the P-values of the fixed factors in the reduced model. Model reduction continued until only significant factors (P < 0.05) and their lower order interaction terms were left (Steyerberg, 2009). As part of the description of spatial distribution of acaricide RR, Moran's I was computed to investigate spatial auto-correlation. It is a measure of spatial autocorrelation that quantifies the similarity in attribute values between neighboring locations. It ranges between −1 and 1, where positive values indicate positive spatial autocorrelation (similar values are clustered together), negative values indicate negative spatial autocorrelation (dissimilar values are clustered together), and values close to zero indicate no spatial autocorrelation. Calculation of the statistic was done on two sample clusters in South Africa (Cluster 1: Longitude 29.0–31.5 and Latitude −28.0 to −31.0; Cluster 2: Longitude 31.0–31.7 and Latitude −24.5 to −26.5; Fig. 1) and 2 clusters in Brazil (Cluster 3: Longitude −58.0 to −50.0 and Latitude −24.0 to −32.0; Cluster 4: Longitude −46.0 to −43.0 and Latitude −21.0 to −15.0) when at least 9 sampling points were available. All data management and statistical analyses were done in SAS v 9.3 (SAS Institute, Cary, North Carolina, USA).

## 3 Results

### 3.1 Macro-geographic variation in phenotypic resistance

Overall, 88.7 % (63) of the 71 investigated isolates showed RR > 3.0 for at least one active ingredient, with a regional variation ranging from 55.6% (lab 2 - Mpumalanga) to 100% (lab 5 - Brazil; Fig. 2 a). Furthermore, multi-resistance in single tick isolates was a common phenomenon in our collection: 69 % of the isolates showed RR > 3.0 for more than one acaricide with a regional variation ranging from 22.2 % (lab 2 - Mpumalanga) to 80 % (lab 4 and 5 - Brazil; See Table 2). Among the resistant isolates, 39.7% showed elevated RR for at least three acaricides (Fig. 2 b). Note that, as for lab 1 (KwaZulu Natal) bio-assays for doramectin and fipronil were not executed, resistance percentages mentioned above are likely to be underestimated.

**Table 2:** Distribution of tick strains over the different levels of acaricide resistance.

Institute	Acaricide (%)	Susceptible (RR <3.0)	Intermediate (3.0–26)	High (>26)	Multi-resistance (N° acaricides RR >3.0)	
Lab 1 – KwaZulu Natal	Amitraz	33.3	50	16.7	**2**	(28.6 %)
	Chlorfenvinphos	60	40	0	**3**	(14.3 %)
	Deltamethrin	33.3	66.7	0	**≥ 2**	42.9 %

Lab 2 - Mpumalanga	Amitraz	100	0	0	**2**	(22.2 %)
	Chlorfenvinphos	50	50	0	**3**	(0 %)
	Deltamethrin	16.7	0	83.3	**4**	(0 %)
	Doramectin	100	0	0	**5**	(0 %)
	Fipronil	100	0	0	**≥ 2**	22.2 %

Lab 3 – Western & Eastern Cape	Amitraz	18.2	72.7	9.1	**2**	(14.3 %)
	Chlorfenvinphos	61.5	38.5	0	**3**	(50.0 %)
	Deltamethrin	0	53.8	46.2	**4**	(14.3 %)
	Doramectin	100	0	0	**5**	(0 %)
	Fipronil	33.3	66.7	0	**≥ 2**	78.6 %

Lab 4 – Brazil	Amitraz	46.4	53.6	0	**2**	(35.5 %)
	Chlorfenvinphos	69	31	0	**3**	(35.5 %)
	Deltamethrin	11.1	77.8	10.3	**4**	(6.5 %)
	Doramectin	96.7	3.3	0	**5**	(3.2 %)
	Fipronil	23.3	73.3	3.1	**≥ 2**	80.6 %

Lab 5 – Brazil	Amitraz	20	70	10	**2**	(70.0 %)
	Chlorfenvinphos	88.9	11.1	0	**3**	(0 %)
	Deltamethrin	0	100	0	**4**	(10 %)
	Doramectin	100	0	0	**5**	(0 %)
	Fipronil	80	20	0	**≥ 2**	80.0 %

Note: for KwaZulu Natal only Amitraz, Chlorfenvinphos, Deltamethrin have been successfully tested.

Resistance ratios (RR) considered to be high (>26), intermediate (3.0–26), or low (<3.0).

Multi-resistance: a tick strain showing intermediate to high resistance to more than one acaricide.

**Fig. 3:**
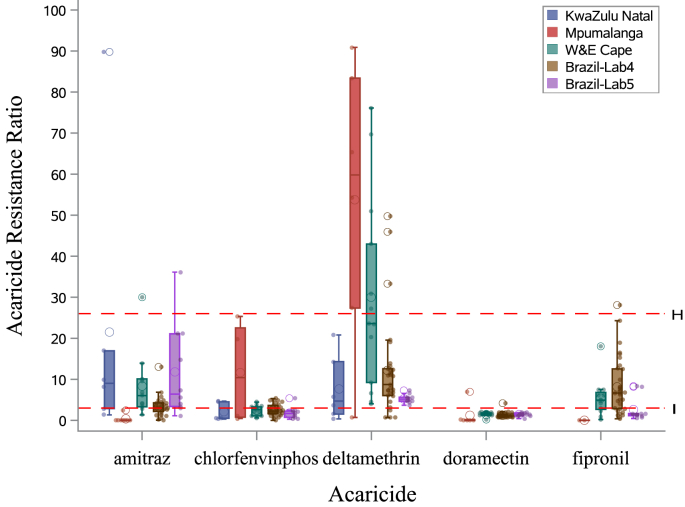
Macro-geographic variation in acaricide RR per acaricide type. Boxplots and raw data (scatter plots) are overlayed, showing the local variation in the isolate collections of each macro-geographic regions. Reference lines are added at RR 3.0 and 26, positioning the ticks that are considered to be susceptible (<3.0), show intermediate (3.0–26.0 “I”) or high resistance (>26.0; “H”) to the active ingredient. Details on collection sites (including the Brazilian labs) are provided in the ‘Materials and Methods’ section.

An overview of the variation in RR is provided in Fig. 3. Substantial acaricide-dependent variation was observed across regions. Highest levels of resistance were observed against deltamethrin in South Africa (lab 3 - W&E Cape, median: 23.58, Coefficient of Variation: 79.42%; and lab 2 - Mpumalanga, med.: 59.8, CV: 64.02%) with ratios up to 90.8 (Mpumalanga) and with considerable within-region variation. One isolate of KwaZulu Natal (lab 1) showed a very high RR against amitraz (89.8). In Brazil, highest (variation in) resistance was observed against amitraz (lab 5, med.: 6.39, CV: 96.61%), fipronil (lab 4, med.: 6.67, CV: 84.97%) and deltamethrin (lab 4, med.: 8.74, CV: 98.15%). Doramectin resistance was low overall, and chlorfenvinphos resistance was relatively low without much variation, except for lab 2 - Mpumalanga (med.: 10.42, CV: 109.53%).

For the calculation of spatial auto-correlation in RR, of the four spatial clusters identified (Fig. 1) the two Brazilian ones, and one South African (Cluster 1: amitraz, deltamethrin only) were considered to be suitable. None of those clusters (with at least 9 observations) showed autocorrelation in RR (range Moran's I: -0.214 to −0.022; all P's > 0.21) for any of the acaricides, indicating that acaricide resistance is spatially not very predictable by its occurrence.

Spearman correlation coefficients, measuring the monotonic association between two acaricide RR (mean-centered at country level for each acaricide) in terms of ranks, showed positive associations between chlorfenvinphos and doramectin (spearman's ρ = 0.43; P = 0.0011; N = 54), chlorfenvinphos and fipronil (ρ = 0.49; P = 0.0002; N = 51), and fipronil and doramectin (ρ = 0.45; P = 0.0007; N = 54) respectively. After removing the effects of potential outlying (extreme) points, correlations remained (ρ = 0.38; ρ = 0.47; ρ = 0.42, respectively; all P's < 0.001; See Additional file 1, Figs. S1–3).

### 3.2 Acaricide resistance and acaricide use

While fipronil resistance was significantly lower on the farms where phenylpyrazole acaricide is currently applied (log-transformed ratios _not used vs used_: 0.45 ± 0.12; Z = 3.80; P < 0.001) the opposite pattern was observed in the past (log-transformed ratios _not used vs used_: -0.82 ± 0.08; Z = −10.49; P < 0.0001; Table 3). Doramectin RR were higher on farms where macrocyclic acaricides are currently used (log-transformed ratios _not used vs used_: -0.22 ± 0.02; Z = 13.71; P < 0.001). In the past, similarly, amitraz RR were higher on farms where amidine acaricides are used (log-transformed ratios _not used vs used_: -1.49 ± 0.01; Z = −19767; P < 0.001). In contrast, chlorfenvinphos RR were lower in farms where organophosphate acaricides have been used (0.55 ± 0.05; Z = 10.66; P < 0.001).

**Table 3:** Associations between acaricide type applied (current and previous use) and resistance ratios.

	Current use	Previous use
**Amitraz resistance**		
*Amidine* (not used vs used)	0.44 ± 0.31	**−1.49 ± 0.01**
	Z = 1.43; P = 0.15	**Z = - 19767; P < 0.001**
	Lab 1, 3	Lab 1,3,5
***Chlorfenvinphos resistance***		
*Organophosphate* (not used vs used)	−0.11 ± 0.06	**0.55 ± 0.05**
	Z = −1.85; P = 0.07	**Z= 10.66; P < 0.001**
	Lab 3,4,5	All except lab 3
***Deltamethrin resistance***		
*Pyretoid* (not used vs used)	−0.45 ± 0.32	−0.03 ± 0.24
	Z = −1.45; P = 0.15	Z = −0.12; P = 0.90
	All	All
***Doramectine resistance***		
*Macrocyclic* (not used - used)	**−0.22 ± 0.02**	−0.03 ± 0.15
	**Z=-13.71; P < 0.001**	Z = 0.05; P = 0.83
	Lab 1,3,4	Lab 4
***Fipronil resistance***		
*Phenylpyrazole* (not used vs used)	**0.45 ± 0.12**	**−0.82 ± 0.08**
	**Z=3.80; P < 0.001**	**Z=-10.49; P < 0.001**
	Lab 4,5	Lab 4,5

**Note:** Log-transformed resistance ratios, in order to meet the assumption of normality.

Effects are show with ±standard error.

Exchangeable working correlation has been included, to take into account the correlated data within the same research institutes.

For each resistance ratio, only the research institutes are included with at least one tick strain that origins from a farm where a certain acaricide type has been used.

See ‘Materials and Methods’ for the description of the labs (Lab 1–5).

For the analyses on fipronil resistance, additional factors that describe the general acaricide use (number of acaricides locally applied, time since last application) explained part of the variation with statistical significance: on farms where more than one acaricide type was used, RR were higher, currently and in the past. The longer it has been since the acaricides have been applied, the lower the local RR (see Table 4).

**Table 4:** Effects of Phenylpyrazole type acaricides on fipronil resistance ratios, as well as effects of general acaricide use (total number of acaricides used, and time since last use) obtained from GEE models using the data of two Brazilian Research institutes (Lab 4 and 5).

	Contrasts	Estimate **±** SE	Z-statistic	P-value
**Current use**				
Intercept		12.74 ± 0.15	8.35	<0.001
Phenylpyrazole	Not used vs Used	**0.46** ± **0.12**	**3.80**	**< 0.001**
Total acaricides	>2 types vs 1 type	**0.62** ± **0.06**	**9.55**	**< 0.001**
	2 types vs 1 type	0.36 ± 0.38	0.97	0.33
Time since last use	>2 months ago vs recent	**−0.20** ± **0.06**	**−3.37**	**< 0.001**
	1–2 months ago vs recent	−0.47 ± 0.17	−2.77	0.006
**Previous use**				
Intercept		23.19 ± 0.25	9.17	<0.001
Phenylpyrazole	Not used vs Used	**−0.82** ± **0.08**	**−10.49**	**< 0.001**
Total acaricides	>2 types vs 1 type	**0.61** ± **0.24**	**2.54**	**0.011**
	2 types vs 1 type	**0.92** ± **0.11**	**8.25**	**< 0.001**
Time since last use	>2 months ago vs recent	−0.21 ± 0.27	−0.78	0.44
	1–2 months ago vs recent	**−0.59** ± **0.04**	**−16.00**	**< 0.001**

**Note:** Log-transformed resistance ratios (in order to meet the assumption of normality).

Exchangeable working correlation has been included, to take into account the correlated data within the same research institutes. Parameter levels for intercept: ‘recently treated farms’ (i.e. ≤ 2 weeks ago) where only one acaricide has been applied.

### 3.3 Link between deltamethrin phenotypic resistance and occurrence of the *kdr*-mutation in the population

#### 3.3.1 Population level data: *kdr*-allele frequencies relationship with phenotype resistance

**Fig. 4:**
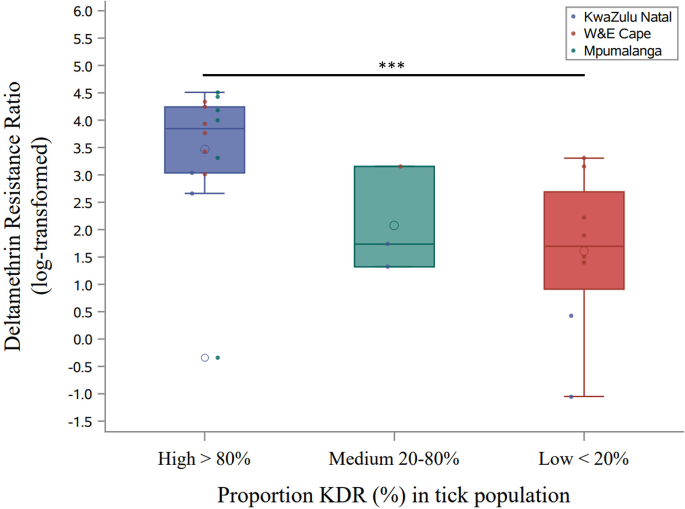
Acaricide phenotype RR in the assays using deltamethrin over the three categories on *kdr*-mutation presence (High, >80%; Medium, 20–80%, and Low, <20%). Boxplots and raw data (scatter plots) are overlayed, further showing the variation within each of the categories. ‘***’ Statistically significant contrast.

Based on the relationship between *kdr*-allele frequencies and phenotypes, isolates for which the *kdr*-mutation was highly prevalent (>80%, N = 14, class ‘High’) showed statistically significantly higher resistance to deltamethrin compared to isolates where the mutations were more rare (<20%, N = 8, class ‘Low’; 37.17 ± 4.21; Z = 8.83; P < 0.001). Nevertheless, in both categories the variation in RR was high. While two isolates categorized in the ‘Low’ class fell well in between the 75 quantile (25%–75%) of the ‘High’ class, there was only one isolate in the ‘High’ class which showed similar level of resistance as observed in the ‘Low’ class. The very few isolates with intermediate *kdr*-prevalence (20–80%; N = 3) fell nearly all below the 25% quantile of the ‘High’ category (Fig. 4).

#### 3.3.2 Individual level data: *kdr*-allele presence in dead and live phenotypes

**Fig. 5:**
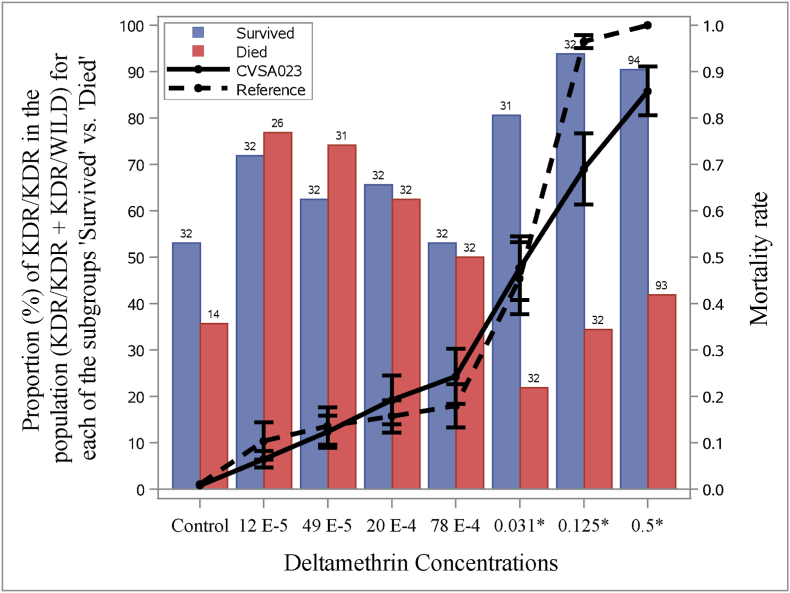
Prevalence of homozygote KDR/KDR in each subgroup (‘Survived’: blue, ‘Died’: red). Batches of ticks from the same isolate (CVSA023) were exposed to one of the deltamethrin concentrations. Overall mortality rate (black line) in CVSA023 and reference isolates (dashed line). Genotypes found in CVSA023 were KDR/KDR (48 %) and KDR/WILD (52 %). The susceptible reference strain (combined mortality rate from three collaborators) were all WILD/WILD (dashed line). Numbers above bars: number of collected larvae genotyped. ‘*’: Statistically significant difference in KDR/KDR proportions between the two subgroups (‘Survived’ and ‘Died’) for a given deltamethrin concentration. (For interpretation of the references to color in this figure legend, the reader is referred to the Web version of this article.)

At the individual level, the *kdr*-mutation presence in the larvae that died and those that survived after exposure to different concentrations of deltamethrin during the LPT was assessed. This was performed in a single population of larvae (stock CVSA023) with a synthetic pyrethroid resistance ratio of 23.45 and for which the population level data for *kdr-*allele frequency was found to be 30%WT/70% *kdr*. This stock was selected to allow for discrimination of allele frequency. For most of the concentration-subgroup combinations, approximately 30 individuals were collected. At the highest concentration, additional individuals were sampled (survived: N = 64; died: N = 63). Given the low mortality rates at the lowest concentration (control group) only 14 dead individuals could be sampled (Fig. 5).

The genotypes found in the population in the control group (i.e. without selection pressure) were ‘KDR/KDR’ (48 %; N = 46) and ‘KDR/WILD’ (52 %). At the three highest concentrations (i.e. when the overall mortality rate was between 0.49 ± 0.05 and 0.83 ± 0.05) among the ticks that survived, the KDR/KDR genotype was more prevalent than the KDR/WILD genotype (χ2 ≥ 21.7; df = 1; P < 0.0001; Fig. 5).

## 4 Discussion

This study has shown a high occurrence of acaricide resistance in *R. microplus* obtained from cattle across vast areas in both South Africa and Brazil. The data presented here provide an overview of resistance from a range of farming practices including commercial and small scale producers. Stocks were collected from locations with and without reports of phenotypic resistance however, given the invasive behavior of the tick (see below) and cattle-assisted transport over long distances, the outcomes are alarming. Furthermore, multi-resistance in single tick isolates was commonly observed, highlighting the significant challenges societies are currently facing with regard to tick control in cattle farming. The global socio-economic importance of *R. microplus* populations (Jongejan and Uilenberg, 2004; Rodriguez-Vivas et al., 2018) has led to extensive exposure to (multiple) acaricides. The short life cycle of *R. microplus* contributes to heavy tick infestations and rapid population growth. Moreover, its competitive advantage, particularly over *R. decoloratus* in Africa, hampers efforts to control the spread and establishment of tick populations, including those resistant to acaricides (Walker et al., 2003; Estrada-Pena et al., 2006). The issue of acaricide resistance is compounded by both farmer-related problems, such as acaricide overuse and misuse, and inadequacies in local control policies like national acaricide protocols and animal movement control. These factors exacerbate the problem of acaricide resistance, especially when target-specific mutations and metabolic adaptations are sufficient to allow ticks to overcome the effects of parasiticides. This can lead to rapid evolution of acaricide resistance, as explained in Rodriguez-Vivas et al. (2018) under the section 'Factors influencing the rate of emergence of resistance to acaricides.

In both South Africa and Brazil, resistant tick isolates are frequently encountered, as demonstrated in this study. While the presented data establishes correlations between acaricide use and resistance, assessing causal relationships remains challenging. Nevertheless, several noteworthy associations at a local scale were observed. Specifically, current resistance to amitraz and fipronil in ticks tends to be higher on farms where their respective acaricide classes, amidine, and phenylpyrazole, have been historically used. This suggests a possible micro-evolutionary response of tick isolates under acaricide selection pressures. Conversely, farms with past organophosphate use exhibited lower chlorfenvinphos resistance. Additionally, fipronil resistance was found to be lower on farms currently employing phenylpyrazoles, indicating successful targeting of susceptible populations. Interestingly, farms facing more significant challenges with fipronil resistance tended to intensify their acaricide use. This was evident through shorter time intervals between successive acaricide treatments and a higher diversity of treatments, likely as a response to combatting the problem. Regarding macrocyclic compounds, doramectin resistance was identified on treated farms (see Table 3). It is worth noting that macrocyclic compounds are also utilized as anti-helminth drugs - although in general less frequently than its use as acaricide - potentially leading to the development of tick resistance that might have gone unnoticed (Molento and Brandao, 2022). It is essential to exercise caution when interpreting the above-mentioned hypothetical time-dependent processes due to the lack of longitudinal data on acaricide resistance, including follow-up data. As a result, further evaluation and research are needed to better understand these complex dynamics.

Multi-resistance to three or more compounds was a common finding (see Table 2 and Fig. 2b). This discovery is profoundly concerning and highlights the significant issue of unintentional selection for acaricide resistance. The problem is compounded by the limited availability of effective chemicals and the uncertainty surrounding the development of new tick control agents. The observed correlations in acaricide resistance ratios may be attributed to shared selection histories among tick populations. However, to the best of our knowledge, there are no indications of common physiological pathways affected by the respective acaricides or a shared genetic basis for their susceptibility. As emphasized by Klafke and colleagues (Klafke et al., 2017), it is of utmost importance to establish and utilize precise diagnostic tools for rapidly detecting these resistant tick populations. Swift identification can help prevent their spread to other areas where this phenomenon has not yet occurred, thus mitigating further challenges.

Special attention was paid to synthetic pyrethroid resistance and its connection with *kdr*-mutations (Cossio-Bayugar et al., 2020). Notably, we observed that deltamethrin resistance in the African collections tended to be higher than in Brazilian ones, setting it apart from other acaricides (Fig. 3). This discrepancy may be due to historical acaricide use leading to local evolution. Selection pressure in Brazil may be relatively lower because of the more frequent use of and easier access to acaricidal drugs. Additionally, restricted gene-flows – driven by human and geographical isolation – may reduce copulations with wildtype susceptible and/or create contexts where the wild type is less competitive in terms of fitness. At the population level (based on data from South Africa), the *kdr*-allele frequencies were positively correlated with deltamethrin RR (Fig. 4). At the individual tick level, the absence of *kdr*-mutations (i.e. WILD/WILD genotypes in all African reference isolates; Fig. 5) was associated with higher susceptibility to the acaricide. The more prevalent the *kdr*-type allele, the less the ticks were susceptible to the acaricide, particularly at higher concentrations. Although we only had one isolate available with individual variation in KDR- and WILD-genotypes (CVSA023) and for which individuals (alive and dead) had been purposely collected, our overall observations indicate that both individuals and populations with *kdr*-mutations are more successful in terms of fitness under selection pressure of a synthetic pyrethroid acaracide. Furthermore, based on previous research, we learned that (multi-) resistance can persist in tick populations for many generations, even in the absence of the respective acaricides, with no significant negative fitness consequences (Rodriguez-Vivas et al., 2018). As point-mutations alone can be sufficient for resistance development, coupled with the substantial reproductive outputs by female ticks, rapid interventions—preferably before resistance is observed—become highly desirable (Klafke et al., 2017).

Hereto, the development of standardized, field-deployable and affordable diagnostics that rapidly detect genetic mutations that are linked to the actual phenotypic resistance, and to which farmers find easy access, would be ideal. Firstly, the test outcomes guide strategy plans nationally to avoid further development of resistance in tick populations. In particular, the generation of data sets on spatio-temporal patterns of acaricide resistance (phenotypic with complementary molecular data) allow for effective modelling of the development of resistance and inform the design and implementation of strategies for combatting this. Secondly, the outcomes enable a more precise estimation of the real burden linked to acaracide resistance and the associated socio-economic damage, which is hard to obtain via phenotypic tests due to the practical limitations (see above). To achieve this, we advocate for coordinated data collection on tick resistance and molecular screening of resistant populations across diverse geographic regions. This approach will enable the early detection of mutations that could compromise test sensitivity. When such changes are detected, primer sequences can be promptly adapted and disseminated among labs and users to ensure that diagnostic kits remain effective and up-to-date. Moreover, the genetic material provided by these screenings will facilitate the sequencing of additional genes, allowing for further assay development and the expansion of molecular markers associated with different acaricide classes. Since multiple genes play a role in overcoming acaricides, we propose utilizing a suite of diverse genetic markers from various enzyme and protein structures targeted by the acaricides. This multivariate data will enable us to fine-tune and optimize intervention policies effectively. We also suggest conducting field validations to progressively increase the number of collaborators and, consequently, the extent of sampling. This collaborative effort will contribute to building a central database to map resistance, which will provide valuable insights and potentially lead to point-of-sale advice on the most effective acaricides and their proper use. By embracing these strategies, we can enhance our understanding of tick resistance and significantly improve intervention strategies to combat acaricide resistance, benefiting both farmers and livestock industries worldwide.

Although an even more standardized study design would have been preferrable, logistical restrictions prevented intercontinental shipping of a reference tick isolate. Nonetheless, the outcomes in this study underscore the importance of the regional spatial variation in resistance, and as a consequence, control measures will likely need to be locally tailor-made. Integration of molecular data together with other sources of information like: local acaricide use, susceptibility of cattle breeds and (transboundary) movement of cattle between regions, together with a good understanding of current socio-economic and climate complexities, will enable policymakers and scientists to provide prevention strategies (Rodriguez-Vivas et al., 2018). All this will optimize the use of financial resources of resource-poor farmer communities, limit the development of resistance, lower the morbidity and mortality in cattle, increase production and animal welfare, and maybe most importantly, will shift land use towards more sustainable agriculture at smaller scales.

## Availability of data and materials

The datasets generated and/or analysed during the current study are available from the corresponding author on reasonable request.

## Authors’ contributions

ML, MM, FM, JF, DJAH and AE conceived and designed the study. DJAH drafted the initial manuscript and executed the statistical analyses. All authors critically reviewed and approved the final manuscript. DJAH, FM, ML, CM, JF and AE analysed the data. GK, LCJ, TS, JW, LM and CS performed field collections and field validation of the assay. All other authors gave substantial scientific input on the final manuscript version. All authors read and approved the final manuscript.

## Declaration of competing interest

The survey was funded by the 10.13039/100000865Bill & Melinda Gates Foundation (BMGF) (Grant OPP1213344) for which LN was a representative. The Sponsor did not dictate study design (but reviewed and approved the protocol), and was not involved in collection, analysis or interpretation of data (but did review and approve the report). Clinglobal and its affiliated companies were contracted (project no. CG1137) to perform the study, and at the time of conduct ML, CM, LM, FM, JF, MM and AE were employed by Clinglobal or its affiliates. GK, LCJ, TS, JW, CS collaborated with Clinglobal to collect tick stocks and perform field validation of the assay.

Declarations of interest: none.
