# Supplementary material for: Phenotypic and genotypic characterization of acaricide resistance in Rhipicephalus microplus field isolates from South Africa and Brazil
Source: Int J Parasitol Drugs Drug Resist. 2023 Dec 27;24:100519. doi: 10.1016/j.ijpddr.2023.100519 (PMC10797136; doi:10.1016/j.ijpddr.2023.100519)
Supplement: Multimedia component 1 [file mmc1.docx]

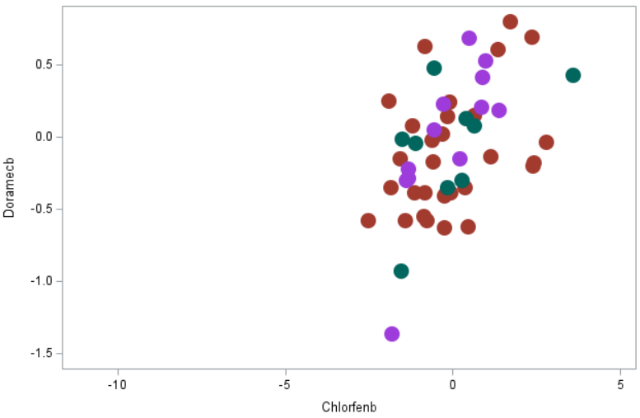

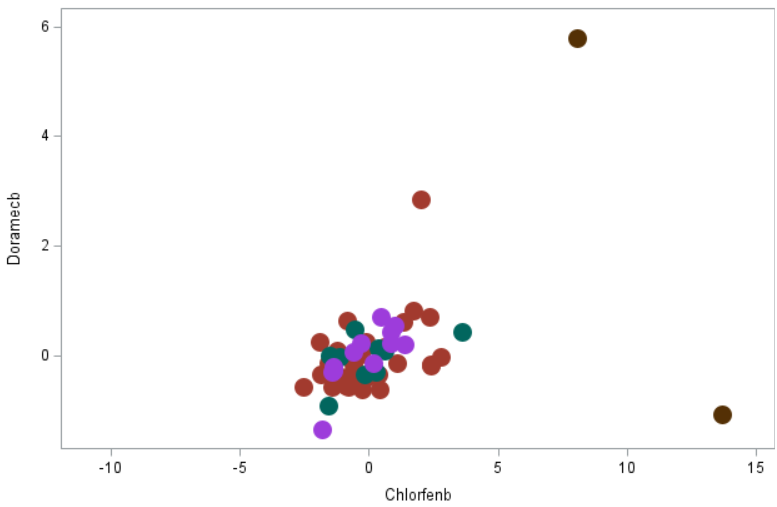

**Fig. 1**


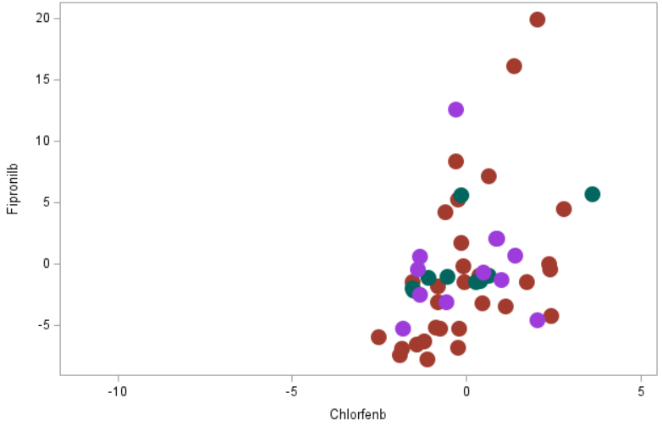


**Fig. 2**


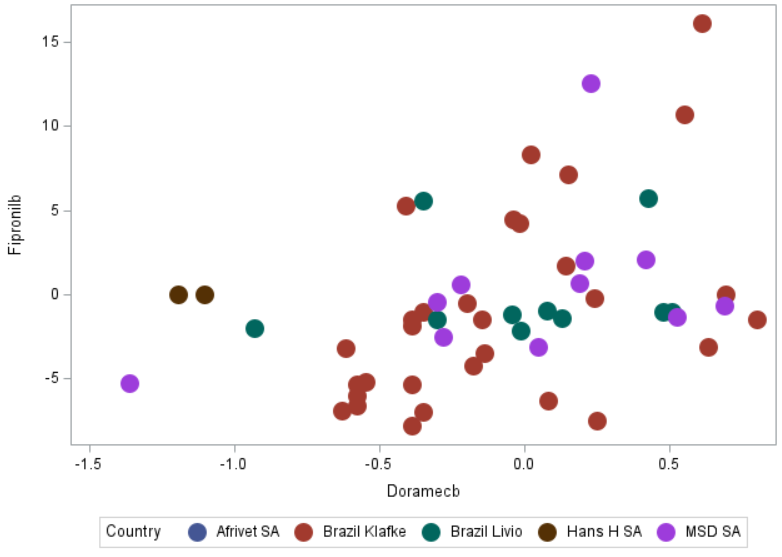

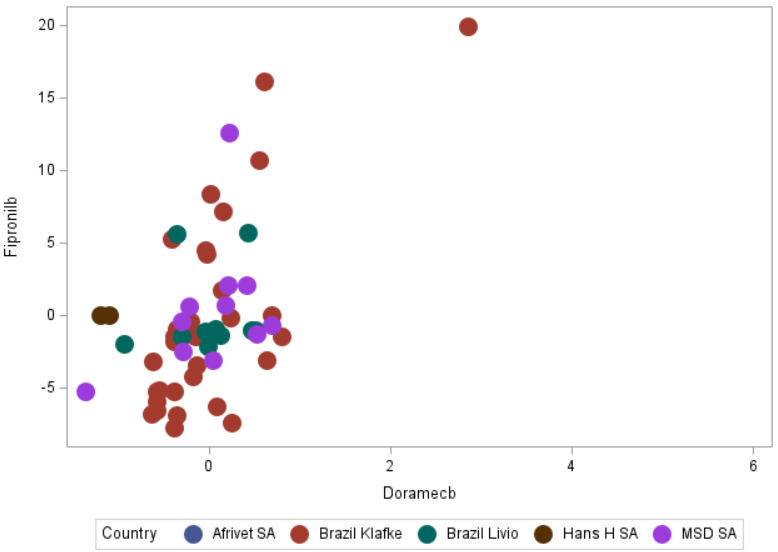


**Fig. 3**

**Figure 1-3:** Scatter plots of mean-centered (at study organization level) acaricide resistance ratios (RR) without (left) and with (right) outlying observations. See main text for spearman rank correlation coefficients.
